# Valley filter and valve effect by strong electrostatic potentials in graphene

**DOI:** 10.1038/s41598-017-10460-5

**Published:** 2017-08-31

**Authors:** Juan Juan Wang, Su Liu, Jun Wang, Jun-Feng Liu

**Affiliations:** 1 School of Physics, Southeast University, Nanjing, 210096 China; 2 Department of Physics, South University of Science and Technology of China, Shenzhen, 518055 China

## Abstract

We report a theoretical study on the valley-filter and valley-valve effects in the monolayer graphene system by using electrostatic potentials, which are assumed to be electrically controllable. Based on a lattice model, we find that a single extremely strong electrostatic-potential barrier, with its strength exceeding the hopping energy of electrons, will significantly block one valley but allow the opposite valley flowing in the system, and this is dependent on the sign of the potential barrier as well as the flowing direction of electrons. In a valley-valve device composed of two independent potential barriers, the valley-valve efficiency can even amount to 100% that the electronic current is entirely prohibited or allowed by reversing the sign of one of potential barriers. The physics origin is attributed to the valley mixing effect in the strong potential barrier region. Our findings provide a simple electric way of controlling the valley transport in the monolayer graphene system.

## Introduction

Recently, the valley transport in 2D graphene-like materials has attracted much attention of researchers, because it is expected that the valley degree of freedom of electrons can exert the same effect as the electron spin in carrying and manipulating information^1–3^. This newly rising discipline is referred to as the valleytronics in a much similar way to spintronics. In graphene, the valley degree of freedom comes from the fact that the six corners of the hexagonal Brillouin zone are divided into two inequivalent groups, labeled as the *K* or *K*′ valley. These two valleys are related by the time-reversal symmetry and can be transformed into each other by spatial inversion operation. Due to much momentum difference between the two valleys, the intervalley scattering is suppressed^4–7^ in clean graphene samples and valley is largely a conserved quantum number in electron transports.

There were tremendous works devoted to the valleytronics field, especially, after several research groups have measured and confirmed valley currents driven by the Valley Hall effect in the monolayer^8^ or bilayer graphene^9, 10^ system. At present, the production and measurement of an imbalance of valley carriers are still the principal tasks in this field, since the valleytronics is still in its infancy. A lot of proposals in the literature were put forward to generate valley-polarized currents by using graphene nanoribbon/nanoconstriction^11, 12^, electromagnetic or optical field^13–21^, and line defects^22–24^, as well as lattice strain^25–34^.

Since graphene has excellent flexibility and the lattice deformation can bring about the opposite pseudo-gauge potential or magnetic field for two valleys^34^, the lattice strain is an ideal method to affecting the valley-dependent transport of electrons. For example, Settnes *et al*.^35^ has recently proposed to use the nanobubble-type lattice deformation in graphene to filtrate and split valleys, and showed that some concrete lattice deformation makes valley polarization of electrons quite high or valley completely splitted in real space. Milovanović and Peeters^36^ studied the same strain-induced bump structure in a graphene ribbon system and found an effective valley filter phenomenon under some special parameters. Certainly, the accurate control of strains in graphene is a great challenge in order to obtain a special pseudomagnetic field. The ideal way of controlling the valley degree of freedom in experiment should be an electric one like that in spintronics.

Given the fact that the valley in graphene is defined in the momentum space with the electron energy around the Dirac points, one can see that the valley definition is no longer valid if the electron energy is far from the Dirac points. i.e., the valley will be severely mixed when the electron transport occurs at this energy level. According to this inference, we study the possible valley-filter and valley-valve effect in monolayer graphene modulated by extremely strong electrostatic-potential barriers, which are assumed to be constructed by gate voltage. It is shown that the potential barrier almost blocks one valley but allows the opposite valley passing through, and the filtering efficiency is quite high even amounting to 100%. The filtered valley is dependent on the sign of the potential barrier and the transport direction of electrons. It is also found that the two opposite potential barriers can bring about an efficient valley-valve effect similar to the GMR effect in the spintronics field. This method by using the electrostatic potentials to controlling valleys does not involve any external magnetic field or material, and is favorable to experiment observation.

## Model and Method

**Figure 1:**
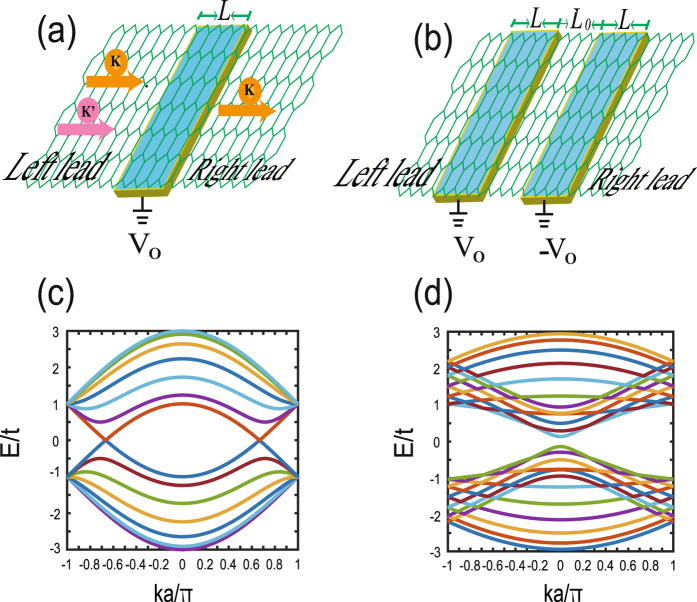
(**a**) Schematic of a two-terminal graphene device with a strong electrostatic potential barrier *V*_0_ constructed by a gate voltage. The barrier can filter *K* or *K*′ valley depending on the sign of *V*_0_ and the electron transport direction. (**b**) Two opposite *V*_0_ barriers are constructed in a monolayer graphene layer consisting of a Valley-Valve device. Energy dispersion *E*-*k* for the zigzag-edge graphene (**c**) and the armchair-edge graphene (**d**) with a periodic boundary condition. The valleys are separated in momentum space for the former case while the valley are degenerate in latter one.

We first consider a simple two-terminal device in Fig. 1(a), where an electrostatic-potential barrier *V*_0_ is constructed in pristine graphene and connects directly with the left and right graphene leads. The barrier length is *L* and its shape is set as a rectangle one, which does not bring any qualitative effect on our results. Since we focus on the valley filter and valve effects, which are induced by the possible valley-mixing effect in the strong potential barrier, a lattice model is employed to describe the system

1 $$H=-t\,\sum _{\langle ij\rangle }\,({c}_{i}^{\dagger }{c}_{j}+c\mathrm{.}c\mathrm{.)}+\sum _{i}\,{V}_{0}{c}_{i}^{\dagger }{c}_{i},$$

where the first term stands for pristine graphene, 〈*ij*〉 denotes the nearest neighboring sites, ${c}_{i}^{\dagger }({c}_{i})$ is the creation (annihilation) operator at the site *i*, and *V*_0_ is the on-site energy representing the barrier region, which is assumed controllable by gate voltages. The spin degree of freedom of electrons is omitted here and the graphene leads are absent of any interaction of electrons. The valley-dependent transmission *T* ^*ττ*′^ at the Fermi energy *E* is given by

2 $${T}^{\tau \tau ^{\prime} }=Tr\,[{{\rm{\Gamma }}}_{L}^{\tau ^{\prime} }{G}_{LR}^{r}{{\rm{\Gamma }}}_{R}^{\tau }{G}_{LR}^{r\dagger }],$$

where *τ*, *τ*′(=*K*, *K*′) is the *K* or *K*′ valley index. *T* ^*ττ*′^ stands for the transmission coefficient of the *τ*′ valley electrons in the left lead that are transformed into the *τ* valley in the right graphene lead. ${G}^{r}={[E-{H}_{c}-{{\rm{\Sigma }}}_{L}^{r}-{{\rm{\Sigma }}}_{R}^{r}]}^{-1}$ is the retarded Green’s function of the device, and *H*_*c*_ is the Hamiltonian of the barrier region, ${{\rm{\Sigma }}}_{L(R)}^{r}$ is the left (right) self-energies of graphene leads with ${{\rm{\Sigma }}}_{L,R}^{r}={{\rm{\Sigma }}}_{L,R}^{rK^{\prime} }+{{\rm{\Sigma }}}_{L,R}^{rK}$ and ${{\rm{\Gamma }}}_{L,R}^{\tau }=i({{\rm{\Sigma }}}_{L,R}^{r\tau }-{[{{\rm{\Sigma }}}_{L,R}^{r\tau }]}^{\dagger })$, i.e., the left and right self-energies consists of the *K* and *K*′ dependent self-energy ${{\rm{\Sigma }}}_{L,R}^{rK}$ and ${{\rm{\Sigma }}}_{L,R}^{rK^{\prime} }$. The Green function ${G}_{LR}^{r}$ is calculated by usual recursion method, while the self-energy of the graphene lead can be constituted by the left- or right- propagating electron eigenfunctions at the Fermi energy. It is assumed that *H*_0_ is the Hamiltonian of a unit cell of the uniform graphene lead, and the hopping matrix between the neighboring cells is *H*_*LR*_ as well as its hermite conjugate *H*_*RL*_ = [*H*_*LR*_]^†^. Then the eigenfunction |*χ*〉 satisfies

3 $$H(k)|\chi \rangle =({H}_{0}+{e}^{ika}{H}_{LR}+{e}^{-ika}{H}_{RL})\,|\chi \rangle ,$$

where *H*(*k*) is the Bloch Hamiltonian, *k* is the wavevector, and *a* is the lattice constant. The energy dispersion of electrons in the graphene lead is worked out by diagonalizing *H*(*k*). One can follow the method in ref. 37 to obtain the left-going (+) or right-going (−) valley-dependent wavefunction $|{\chi }_{m}^{\tau }(\pm )\rangle$ at the Fermi energy *E* with its corresponding wavevector ${k}_{m}^{\tau }(\pm )$, where *m* = (1, …, *M*) and *M* is the matrix dimension of the unit cell *H*(*k*). Afterwards, the left and right propagation matrices *F* ^*τ*^(±) can be directly constructed as

4 $${F}^{\tau }(\pm )={U}^{\tau }(\pm )\,(\begin{array}{ccc}{e}^{i{k}_{1}^{\tau }(\pm )a} &  & \\  & \ddots  & \\  &  & {e}^{i{k}_{M}^{\tau }(\pm )a}\end{array})\,{[{U}^{\tau }(\pm )]}^{-1},$$

where ${U}^{\tau }(\pm )=({\chi }_{1}^{\tau }(\pm ),\ldots ,{\chi }_{M}^{\tau }(\pm ))$ is a matrix from the eigenfunction $|{\chi }_{m}^{\tau }(\pm )\rangle$ at the Fermi energy *E*. According to these propagation matrix, one can build directly the valley-dependent self-energies of the left and right leads as

5 $${{\rm{\Sigma }}}_{L}^{\tau }={H}_{RL}{[{F}^{\tau }(-)]}^{-1},$$

and

6 $${{\rm{\Sigma }}}_{R}^{\tau }={H}_{LR}{F}^{\tau }(+).$$

The electron transporting is assumed along the zigzag edge of graphene as shown in Fig. 1(a), because in this case, the wavefunctions of electrons are clearly valley-separated, i.e., the propagating wavevectors of two valleys are different and one can easily construct the valley-dependent self-energy of leads. Notice that we take an periodic boundary condition along the transverse direction in order to avoid the zero-edge state in the zigzag nanoribbon system and simulate a very large graphene system. The energy band of the graphene lead is shown in Fig. 1(c), where the two Dirac points explicitly denotes two valleys at *K* = 2*π*/3*a* and *K*′ = −2*π*/3*a*. At *E* > *t*, there is no clear definition of valley and thus, the valley-dependent scattering will occur. The energy diagram of the armchair-edge graphene is also plotted in Fig. 1(d), where the valley is shown degenerate around Dirac points. This implicates that the valley transport should heavily depend on the propagation direction of electrons in the graphene lattice, e.g., along the zigzag-edge or the armchair edge. The *K*-valley (*K*′-valley) transmission of electrons is defined as *T* ^*K*^ = ∑_*τ*_ *T* ^*Kτ*^ (*T* ^*K*′^ = ∑_*τ*_ *T* ^*K*′*τ*^), so the valley filtering efficiency can be represented by the dimensionless transmission as

7 $${\eta }_{K({K}^{^{\prime} })}=\frac{{T}^{K({K}^{^{\prime} })}}{{T}^{K}+{T}^{{K}^{^{\prime} }}}.$$

A valley-valve device similar to the spin-valve one is also considered as schematically shown in Fig. 1(b), where two opposite potential barriers are put onto the monolayer graphene, which may be regarded as an antiparallel configuration. Similarly, the device is termed as the parallel configuration when the two potential barriers have the same sign. The valley-valve efficiency (*vve*) is defined as the difference between the conductances of these two configurations.

8 $$vve=\frac{{T}_{p}-{T}_{ap}}{{T}_{p}+{T}_{ap}},$$

where *T*_*p*,*ap*_ = *T* ^*K*^ + *T* ^*K*′^ is the total transmission of electrons in the parallel (antiparallel) structure of the valley-valve device. Certainly, *vve* should critically depend on the efficiency of the valley filtering effect in a single barrier region which is relied on *V*_0_.

## Results and Discussions

In our calculations, we take the hopping energy *t* = 1 as the energy unit, the temperature as zero *T* = 0 K, and the Fermi energy as *E* = 0.1*t*. The transverse width of the device is set as 2048 atoms in a unit cell amounting to 220 nm or so with the lattice constant *a* = 2.44 Å.

**Figure 2:**
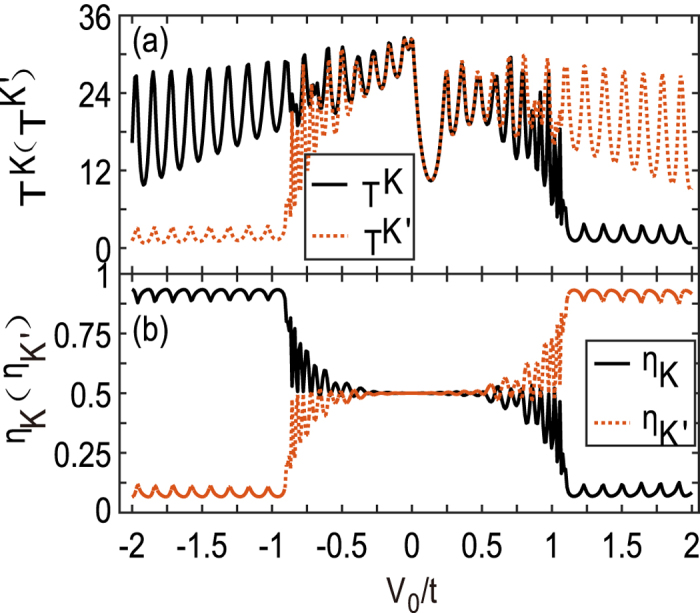
*K* and *K*′ valley-dependent transmission coefficients *T* ^*K*^(*T* ^*K*′^) (**a**) and the valley filter efficiency *η*_*K*(*K*′)_ versus the barrier strength *V*_0_. The length of the two-terminal device is *L* = 20*a* and the Fermi energy is *E* = 0.1*t*.

We first present the electron transmission in the simple two-terminal device in Fig. 2(a), where *T* ^*K*^ and *T* ^*K*′^ is plotted as a function of the barrier strength *V*_0_. It is shown that the electrostatic potential ${V}_{0}/t\mathop{ < }\limits_{ \tilde {}}0.2$ near the Dirac point *E* = 0 does not lead to serious valley splitting of the electron transmissions. When |*V*_0_| is far from the Dirac point significantly, the valley splitting becomes conspicuous. At $|{V}_{0}|/t\mathop{ > }\limits_{ \tilde {}}1$, one valley transmission exceeds plumb the other one. As a result, the device functions as a valley filter and this is clearly shown in Fig. 2(b). The valley filter efficiency can amount to as high as ${\eta }_{K({K}^{^{\prime} })}\sim \mathrm{94 \% }$. The clear oscillations come from the resonant transmission of electrons through the rectangle potential barrier *V*_0_ region.

In Fig. 2, the efficient valley filtering effect becomes evident just at $|{V}_{0}|/t\mathop{ > }\limits_{ \tilde {}}1$ and keeps almost unchanged afterwards. This stems from the fact that when the electron energy meets *E* > *t* in Fig. 1(c), the valley degree is no longer valid, and more importantly, the transport modes are shrunk by half because of no valley degeneracy. This immediately indicates that one valley species of electrons incident from leads will be blocked, which is certainly determined by the wavevector match. Therefore, for the electrons incident from the opposite graphene leads, the opposite valley is blocked. This is also the requirement of the time-reversal symmetry. In addition, for a weak *V*_0_, there is no valley (mode) shrinkage so as to no valley filter effect.

Since our studied system has the time-reversal symmetry, we have ${T}^{K}({V}_{0},E)={T}^{{K}^{^{\prime} }}(-{V}_{0},-E)$ and *T* ^*ττ*′^ = *T* ^*τ*′*τ*^. The curves are not rigorously symmetric upon *V*_0_ = 0 in Fig. 2, because the Fermi energy *E* = 0.1*t* is fixed in numerics. Certainly, for the strong barrier case ${V}_{0}\gg E$, the symmetry is recovered. Moreover, the opposite sign of potentia barrier *V*_0_ will lead to opposite filtering effect. In other words, reversing the sign of *V*_0_ will lead to the opposite valley filter effect. Additionally, the *K* and *K*′ valley electrons have the opposite wave vectors, so the left-going *K*′ valley would be blocked by the potential barrier *V*_0_ if the right-going *K* valley is prohibited by the same barrier potential.

**Figure 3:**
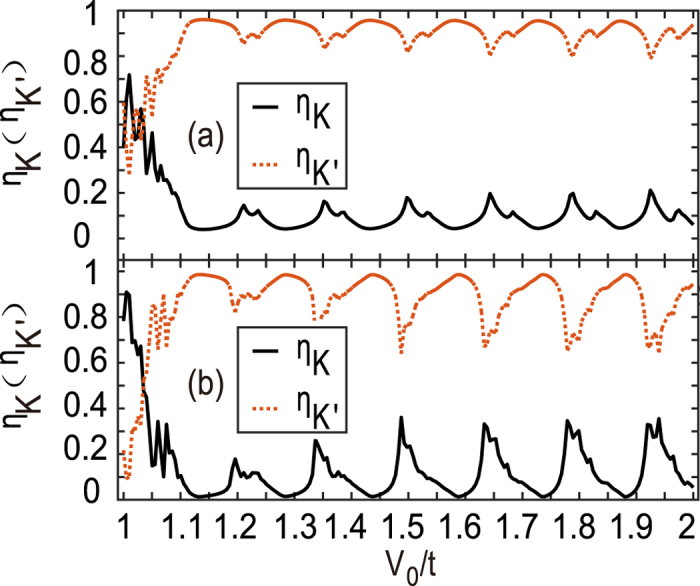
Valley filter efficiency *η*_*K*(*K*′)_ versus the barrier strength *V*_0_ for the two-barrier (**a**) and four-barrier (**b**) superlattice device. All barriers are assumed to be the same, and the barrier length and the distance between two neighboring barrier are *L* = *L*_0_ = 20*a*.

The single potential barrier *V*_0_ can filtrate valley and the efficiency is quite high, but it is not perfect, since the intervalley scattering due to *V*_0_ cannot be depressed entirely and the symmetry of *T* ^*ττ*′^ = *T* ^*τ*′*τ*^ persists. A superlattice structure consisting of multiple *V*_0_ barriers should in principal enhance the efficiency further. In Fig. 3, the two- and four-barrier superlattice devices resembling that in Fig. 1(b) are studied and *η*_*K*(*K*′)_ is shown. One can see that the overall efficiency *η* is enhanced and the maximum efficiency even arrives at 100% in the case of the four-barrier device in Fig. 3(b). This is attributed to the resonant tunneling effect. Certainly, the dips in those curves are also strengthened. The curves are not as smooth as ones in Fig. 2, because several different oscillating periodicities superposition together from either the barrier length, *L*, or the distance between the two barriers, *L*_0_.

**Figure 4:**
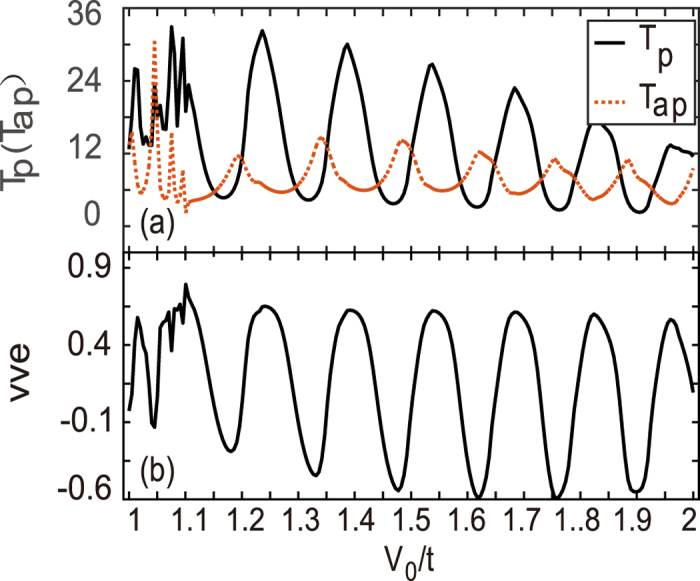
Transmission coefficients *T*_*p*_(*T*_*ap*_) (**a**) and the valley-valve effect *vve* as a function of *V*_0_. The two barriers are the same in the parallel configuration whereas they are opposite in the antiparallel configuration. The length of barrier and the interval distance is set as *L* = *L*_0_ = 20*a*.

Based on the above results, it is naturally envisaged that two opposite barriers consisting in a double-barrier device should block the current totally, which is similar to the GMR effect. This opposite barrier structure can be dubbed as the antiparallel configuration, while the two same barriers *V*_0_ is the parallel configuration. The latter structure shall allow one valley electrons flowing and thus, its conductance should be much sizable in comparison to the former one. Similar to the spin-valve effect, we calculate the conductances of these two configurations and present the valley-valve efficiency *vve* in Fig. 4. It is shown that the transmission *T*_*p*_ of the parallel-configuration device is mostly larger than that of the antiparallel one (*T*_*ap*_) in Fig. 4(a). At some resonances, however, *T*_*ap*_ exceeds *T*_*p*_, and the valley-valve effect is not ideal as expected. This is clearly shown in Fig. 4(b). The exact reason is the coherent transport and the resonant tunneling, which accounts for such oscillating behaviors.

**Figure 5:**
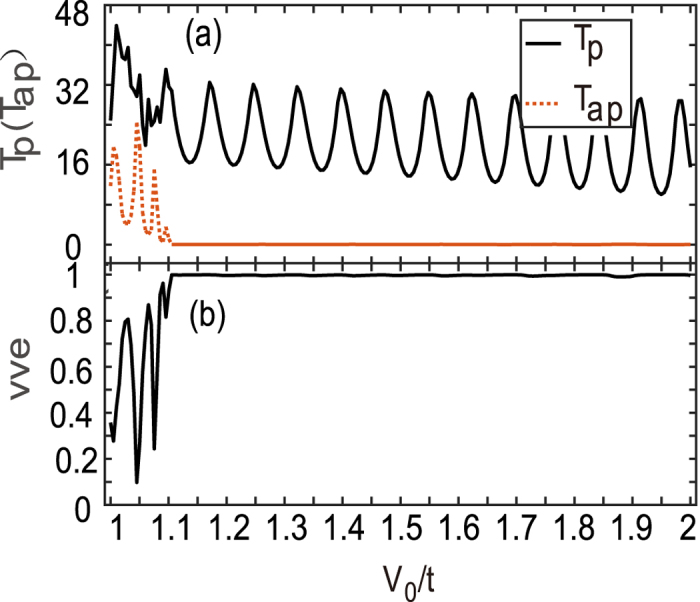
Transmission coefficients *T*_*p*_ (*T*_*ap*_) (**a**) and the valley-valve effect *vve* (**b**) as a function of *V*_0_. Parameters are the same as those in Fig. 4 except *L*_0_ = 0.

In a realistic device, there generally exist some interactions like disorders and electron-phonon coupling, which will smear the phase-coherent transport and may improve the valley-valve effect provided that the intervalley scattering does not severely occur in the process of the incoherent scattering. In our calculations, we simply consider a zero distance between two opposite barriers of Fig. 1(b) (*L*_0_ = 0) in order to diminish the resonant tunneling effect. The results are shown in Fig. 5, where the parameters are the same as those in Fig. 4 except for *L*_0_ = 0. It is shown that *T*_*p*_ > *T*_*ap*_ is valid in nearly all *V*_0_ range (${V}_{0}/t\mathop{ > }\limits_{ \tilde {}}1$), *T*_*ap*_ are entirely suppressed in Fig. 5(a), which in turn leads to a saturated valley-valve efficiency *vve* = 1 in Fig. 5(b).

**Figure 6:**
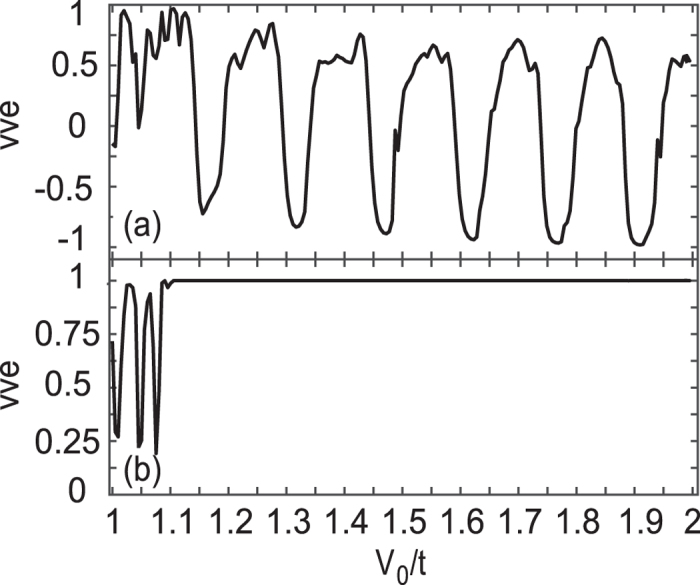
Valley-valve effect as a function of *V*_0_ in a four-barrier superlattice structure for *L*_0_ = 20*a* (**a**) and *L*_0_ = 0 (**b**). Other parameters are the same as those in Fig. 5.

We also consider a four-barrier superlattice structure as that studied in Fig. 3(b), which is actually a parallel structure. While the antiparallel configuration is defined as *V*_0_/−*V*_0_/*V*_0_/−*V*_0_ with an alternative *V*_0_ serial. Both nonzero and zero *L*_0_ cases are calculated and shown in Fig. 6(a,b). Similarly, the efficiency *vve* shows a strong resonant tunneling feature when *L*_0_ is nonzero in Fig. 6(a), i.e., the conductance of the parallel structure does not always exceed the antiparallel one and the peaks and dips are strengthened. Whereas for the case of *L*_0_ = 0, a saturated value *vve* = 1 appears again at ${V}_{0}/t\mathop{ > }\limits_{ \tilde {}}1.1$ in Fig. 6(b), i.e., the current is totally blocked in the antiparallel structure, which is the same as that in Fig. 5(b) again.

In above numerics, the rectangle profile of the potential barrier was employed, however, other continuous and smooth functions of *V*_0_ were also computed but showed no qualitative influence on our obtained results. Actually, a real factor influencing the filtering effect is the transport direction of electrons. As stated earlier, the electron transport is assumed along the zigzag edge and there is a valley (mode) shrinkage phenomenon at *E* > *t* as shown in Fig. 1(c), which is necessary to bring about the valley filter and valve effect. When the transport direction along the armchair edge is considered, the valley is almost degenerate whatever *V*_0_ is taken, and there shall be no valley-filtering or valley-valve effect. Since the doping level of pristine graphene was proved to be changed easily by gate voltages^38^, it is not difficult to observe our proposal of the valley-filter or valley-valve effect.

## Conclusion

In summary, we have proposed a simple method to filter valley in the monolayer graphene system by introducing an extremely strong potential barrier. It is shown that the potential barrier can block one valley but allow the opposite valley tunneling through it, which is dependent on the sign of the barrier as well as the current direction. The valley filtering efficiency can be further enhanced in a multi-barrier superlattice structure. The valley-valve effect was also studied and the device conductance can be significantly controlled by reversing the barrier sign, when the distance between barriers are short enough for suppressing the resonant tunneling effect. These findings are dependent on the electron transport being along the zigzag edge direction of the graphene lattice. Our findings may shed light on fully electric controlling of the valley transport in graphene.
